# Identification of Alzheimer's EEG With a WVG Network-Based Fuzzy Learning Approach

**DOI:** 10.3389/fnins.2020.00641

**Published:** 2020-07-21

**Authors:** Haitao Yu, Lin Zhu, Lihui Cai, Jiang Wang, Jing Liu, Ruofan Wang, Zhiyong Zhang

**Affiliations:** ^1^School of Electrical and Information Engineering, Tianjin University, Tianjin, China; ^2^Department of Neurology, Tangshan Gongren Hospital, Tangshan, China; ^3^School of Information Technology Engineering, Tianjin University of Technology and Education, Tianjin, China; ^4^Department of Pathology, Tangshan Gongren Hospital, Tangshan, China

**Keywords:** Alzheimer's disease, EEG, TSK fuzzy model, weighted visibility graph, feature select, multiple network

## Abstract

A novel analytical framework combined fuzzy learning and complex network approaches is proposed for the identification of Alzheimer's disease (AD) with multichannel scalp-recorded electroencephalograph (EEG) signals. Weighted visibility graph (WVG) algorithm is first applied to transform each channel EEG into network and its topological parameters were further extracted. Statistical analysis indicates that AD and normal subjects show significant difference in the structure of WVG network and thus can be used to identify Alzheimer's disease. Taking network parameters as input features, a Takagi-Sugeno-Kang (TSK) fuzzy model is established to identify AD's EEG signal. Three feature sets—single parameter from multi-networks, multi-parameters from single network, and multi-parameters from multi-networks—are considered as input vectors. The number and order of input features in each set is optimized with various feature selection methods. Classification results demonstrate the ability of network-based TSK fuzzy classifiers and the feasibility of three input feature sets. The highest accuracy that can be achieved is 95.28% for single parameter from four networks, 93.41% for three parameters from single network. In particular, multi-parameters from the multi-networks set obtained the best result. The highest accuracy, 97.12%, is achieved with five features selected from four networks. The combination of network and fuzzy learning can highly improve the efficiency of AD's EEG identification.

## Introduction

Currently, Alzheimer's disease (AD) is becoming a common and serious disease due to organic neurodegenerative and progressive lesions in the brain. The patients always show some typical clinical presentations, particularly in the aspect of cognitive dysfunction such as deficient episodic memory and disabled remembering (Smailovic et al., 2018). The clinical diagnosis of AD currently adopts scale assessment, such as Mini-mental State Examination (MMSE), Montreal Cognitive Assessment (MoCA), activities of daily living (ADL), and physiological detection of cerebrospinal fluid. Patients with severe AD can be observed to have changes in brain structure, such as encephalatrophy, through brain functional imaging. Yang et al. applied magnetic resonance imaging (MRI) to detect the cerebral changes of blood flow and oxygenation in AD and mild cognitive impairment (MCI) subjects, and showed its powerful ability to distinguish from normal controls (Yang et al., 2010). Hiroshi's study has demonstrated progression of atrophy mapping upstream to Braak's stages of neurofibrillary tangle deposition in AD. The main cause of organic brain lesions in AD is considered to be the loss of neurons and synapses (Brenner et al., 1988). It has been suggested that the loss of both synapses and neural pathways leads to a decrease in brain functional connectivity and influences electrical signals of the brain, so it is feasible to diagnose neurotic disease by electroencephalogram (EEG). EEG, which can measure the brain's voltage fluctuations with high temporal resolution, contains plenty of physiological information, and there is growing evidence that EEG may contribute to early recognition of AD patients.

The conventional EEG visual inspection is one of methods widely used in neurological assessment. Numerous previous studies have reported the disappearance of alpha EEG activities, particularly in posterior brain regions, through unaided viewing (Matsuda, 2013; Wang et al., 2015; Horvath et al., 2018). It has also been reported that visual EEG scores of ADs show a strong correlation with dementia severity (Kowalski et al., 2001). In the study of de Waal et al. (de Waal et al., 2011), AD patients with early onset are more likely to show severe diffuse slowing characteristic than those with later onset, which is consistent with the clinical manifestations of AD. In addition, studies have quantified the complexity of electrophysiological activities and reported declined complexity of EEG in AD patients (Cao et al., 2016). The change on the AD brain is also reflected in the perturbations of EEG synchronization. As EEG signals are irregular and non-stationary complex signals, traditional visual inspection is not sufficient for AD EEG identification (Buzsaki and Draguhn, 2004; de Waal et al., 2011; Cao et al., 2016). To address this issue, complex network theory is introduced into AD diagnosis, which aims to describe human brain from a global perspective (Palop et al., 2006; Nimmrich et al., 2015; Cao et al., 2016; Gao et al., 2019).

Over the past few years, more and more researchers have begun to adopt the attractive idea of using complex network methods to characterize the dynamic features of complex systems (Zou et al., 2019). This novel approach is the thorough combination of two frontier research fields, analysis methods of non-linear time series (Hively et al., 2000; Costa et al., 2002, 2005) and complex networks theory (Brown et al., 2004; Boccaletti et al., 2006). Zhang et al. have constructed complex networks with strength of temporal correlation between time series and reported that the behavior information (chaotic or fractal) of time series directly correlate with the topological structures (Zhang and Small, 2006). As an effective tool to get insight into the brain function, the brain network analysis has been widely applied in AD research. The healthy brain was found to work with network properties such as small-worldness, hubness, and rich-clubs, while the AD brain operated with less optimal network topologies (Meunier et al., 2010; Blinowska and Kaminski, 2013; Martijn and van den Heuvel, 2013; Wang et al., 2014, 2016; Deng et al., 2015). Loss of small-world features (toward random network topology) can be observed in functional network constructed from EEG and functional magnetic resonance imaging data (Stam et al., 2007; He and Evans, 2010; Tahaei et al., 2012; Reid and Evans, 2013). Numerous EEG studies have consistently demonstrated decreased functional connections in the higher frequency bands of AD patients compared to controls (Tijms et al., 2013; van Straaten et al., 2014).

Compared to other approaches of constructing complex networks through time sequence, visibility graph (VG) algorithms can better integrate the basic features of time series. Lacasa et al. and Liu et al. converted time series into graphs and extracted the topological features using graph theory methods (Lacasa et al., 2008; Liu et al., 2010). They pointed out that the irregularity of time sequence can be characterized by the network topology. For instance, the periodic sequence can be transformed into regular lattice, while the chaotic series corresponds to random graphs. Subsequent researches began to introduce VG method into the EEG study of neurological diseases, and found features extracted from VG networks can be effectively used as mathematical markers in neurodegenerative diagnosis. VG algorithm was first applied in related research in AD by Ahmadlou et al. They reported that complexity of EEGs computed by VGs can be used in the distinguishing between AD and control EEGs (Ahmadlou et al., 2010).

The VG can only express the existence of edges between different time nodes, but not the strength of the edges. Therefore, Supriya et al. have proposed to combine the weighted edge with the horizontal visibility graph, which are not applicable to all complex network graphs (Supriya et al., 2016). Addressing the limitations of above approaches, Zhu et al. have improved the weighted visibility graph (WVG) algorithm by specifying radian function as the criterion for calculating edge weights in all kinds of complex network, and obtained promising results in the detection of epilepsy (Zhu et al., 2014). Also, studies have shown that the visualization method can preserve the characteristics like reduction of complexity (Polikar et al., 2007; Czigler et al., 2008) and slowing of rhythm (Dauwels et al., 2011; Cao et al., 2015; McBride et al., 2015) in patients with AD. WVG networks retain more structural information of the time series, which is more conducive for AD identification, compared to connectivity networks. Therefore, we apply the WVG method on the feature extraction of Alzheimer's disease. A variety of different parameters are extracted from the visibility graph, and used to further investigate which parameter can be used for diagnosing AD.

After quantitative analysis of complex WVG networks, the valuable information about the time series has been extracted. The machine learning generally approaches the extracted features for training the model and then applies them in signal detection. Traditional machine learning methods, including decision tree, random forests, k-nearest neighbor (KNN), Naive Bayes (NB), logistic regression, and so on (Siegelmann and Holzman, 2010; Hramov et al., 2019), have been widely used in the detection of neurological diseases. However, for systems with highly non-linear characteristics, models that built based on these methods do not characterize real models and be utilized in classification well. With this consideration, a rule-based fuzzy model is proposed and has been widely used in many fields like computer vision, natural language processing, and enhanced learning, achieving remarkable results (Gu et al., 2017). Takagi-Sugeno-Kang (TSK) method is proposed to build a model established by using fuzzy mathematics language to describe some characteristics and internal relations of fuzzy phenomena. Compared with traditional classifiers that lack transparency, TSK can be used in multiple features classification and shows a superior model interpretability, which is defined as the ability to better understand the decision strategies of response functions in a human-interpretable manner in order to interpret internal relationships (Deng et al., 2018). In current applications of machine learning, such interpretability has received wide attention and is considered to be crucial.

In this paper, multiple networks are constructed based on multi-channel EEG, with each EEG channel able to be transformed into one-layer network. Then a number of different network features are extracted from them, which is too much for input feature vectors. In order to explore this problem, some feature selection approaches will be utilized to choose features, and the influence of different screening methods on the final classification results will also be tried. The parameters will be divided into three groups—single parameter from multi-network, multi-parameter from single network, and multi-parameter from multi-network—to observe the difference between the classification results of fuzzy models trained with different types of features. The structure of rest paper is as follows: section Methods and materials is devoted to describing the experimental design, including data collection and subject condition. Meanwhile, the principle of mathematical graph methods and Takagi-Sugeno-Kang (TSK) model adopted in paper are also explained in this part. In section Experimental Results, we performed a statistical analysis of the results and implemented AD recognition based on the proposed framework. Section Conclusion and Discussion includes a discussion of the application and advantages of the proposed model, as well as future work.

## Materials and Methods

### Subjects and EEG Recordings

EEG recordings are collected from AD subjects and control subjects, respectively. The AD group included 30 confirmed AD patients who are diagnosed with mini-mental state examination (MMSE) scores are between 12 and 15. The diagnosis results meet the National Institute on Aging-Alzheimer's Association criteria. All of them have not used antipsychotic drugs, antidepressants, dopamine blockers, or excessive amounts of alcohol, and don't have other neurological or psychiatric disorders or any other serious illness. The AD group includes 18 females and 12 males, whose ages range from 74 to 78. The control group consisted of 30 healthy subjects of matched ages, ranging from 70 to 76 years old, and includes 10 females and 20 males. The MMSE scores of them are between 28 and 30. In order to avoid the impact on EEG activity, all subjects will be prohibited from using neuroactive drugs before the experiment. The data adopted in this paper is from our previous study (Wang et al., 2016), which is approved by the Ethics Committee of Tangshan Gongren Hospital and was conducted in accordance with the Declaration of Helsinki. In addition, all the subjects in this experiment gave informed consent.

A 16-channel EEG monitoring system (Solar2000B) is adopted. The EEG channels have 10 MΩ input impedance with bandwidth as 0.08–300 Hz. In order to obtain low-frequency signals that meet the analysis requirements, the low-pass filtering range is set to 0.08–50 Hz. Studies have demonstrated that the EEG amplitude across different bands tends to stabilize when the scalp-electrode impedance is <10 kΩ, so electrode impedance in our experiments is set to 3 kΩ. The international 10–20 system, which consists of 16 electrodes, is adopted as electrode distribution in the scalp (surface) EEG recordings, and the linked earlobe A1 and A2 are used as reference. EEGs are recorded by Symtop amplifier (model: UEA–B; frequency: 1,024 Hz; electrode impedance: 3 k).

During the experiment, the subjects stayed in a semi-dark quiet room and were told to keep awake with eyes closed. The EEG recording process was kept to at least 30 min for each subject. In order to eliminate the impact of nervousness, anxiety, and head movement, a 10-min EEG is selected from each recorded EEG epoch. Sharp transient artifacts caused by eye movement and muscle artifacts, as well as segments with voltage exceeding 150uV, are also removed. Next, fifteen epochs without artifacts with an 8-s long duration for each (15 ^*^ 8 s = 120 s) were chosen for each subject's EEG, which are suitable for weighted visibility graph construction.

### WVG Methods

**Figure 1:**
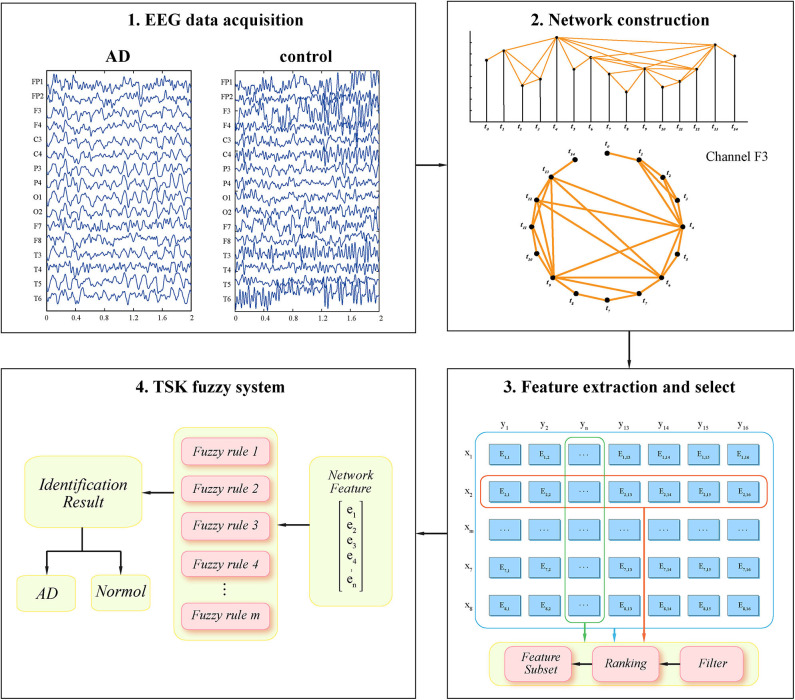
The framework of our method for classifying the AD patients in EEG signals. First multichannel EEG signals of two types of subjects are acquired and a preliminary analysis was performed. Second, we construct the WVG network based on each EEG channel. Third, the features are extracted and further ranked based on feature select method. Finally, we combine the network theory with a fuzzy rule-based system to identify AD pattern with the selected network topological properties.

The EEG signal is the electrical signal of the brain neurons measured on the surface of the cerebral cortex or scalp. It has obvious non-stationary, non-linear, and dynamic characteristics. The VG method provides a way to research the underlying dynamics of EEG data (Lacasa et al., 2008; Deng et al., 2018). Since the VG can inherit the dynamic nature of creating time series data, this technique has the characteristics of describing time series from the perspective of graph theory. The VG algorithm was originally applied in the field of robot motion planning, architectural design, and topographic descriptions of geographical space (Lozano-Pérez and Wesley, 1979; Turner et al., 2001; Lacasa et al., 2009; Jiang et al., 2017; Zou et al., 2019). This algorithm combined the mutual visible relationship of the point and obstacles in the two-dimensional landscape with the computational geometry framework. The literature study reveals that WVG can also be used in EEG data analysis to convert non-stationary, one-dimensional time series into two-dimensional viewable views for analysis. Different channels of EEG signals can reflect the electrophysiological information from different regions of the brain, so each single channel can obtain single complex network and multi-layer networks can be obtained through multi-channel EEG. The schematic diagram of constructing brain network by WVG method is shown in Figure 1.

In the construction of a WVG from a univariate EEG data ${\left\{x_{i}\right\}}_{i=1}^{N}$ with *x*_*i*_ = *x*(*t*_*i*_), individual observations are considered as vertices. Thus, the weighted adjacency matrix **W** can be obtained with size of *N* × *N*. Nodes of WVG network are defined by time points {*t*_*i*_}, *i* = 1, 2, ......*N* and each edge in this network is defined by the connection between two time points (Zou et al., 2019). Two nodes are defined as connected if the criterion

(1) $$\begin{array}{l} \frac{x\left(t_{i}\right)-x\left(t_{k}\right)}{t_{k}-t_{i}}>\frac{x\left(t_{i}\right)-x\left(t_{j}\right)}{t_{k}-t_{i}} \end{array}$$

is fulfilled for all time points *t*_*k*_ with *t*_*i*_ < *t*_*k*_ < *t*_*j*_. Then the absolute value of edge weight between two nodes are determined as

(2) $$\begin{array}{l} w_{i,j}=\arctan\frac{x\left(t_{i}\right)-x\left(t_{j}\right)}{t_{i}-t_{j}},i<j \end{array}$$

### Feature Extraction and Select

The topology of the network is quantified based on the multiple complex networks obtained with WVG method. In order to statistically analyze the characteristics of AD networks and control networks, we calculate the clustering coefficient, average weighted degree, graph index complexity, network entropy, degree distribution index, modularity, local efficiency, and average path length as eight different topological characteristics.

#### Clustering Coefficient

The clustering coefficient is a measure to quantify how tightly connected the neighbor is around a node (Rubinov and Sporns, 2010). For a network *G* with *N* nodes, the connectivity between nodes *i* and *j* is *a*_*i,j*_ (*a*_*i,j*_ = 1 if the connection exists or *a*_*i,j*_ = 0 if not), the weight of connection are *w*_*i,j*_ (*w*_*i,j*_ ∈ [0, 1]). For a weighted network, the local clustering coefficient of node *i* is defined as:

(3) $$\begin{array}{l} C\left(i\right)=\frac{1}{s_{i}\left(K_{i}-1\right)}\underset{j,h\in G}{\sum }\frac{\left(w_{i,j}+w_{i,h}\right)}{2}a_{i,j}a_{i,h}a_{j,h} \end{array}$$

where *s*_*i*_, the strength of the node *i*, is defined as:

(4) $$\begin{array}{l} s_{i}=\underset{j\in G_{i}}{\sum }w_{i,j} \end{array}$$

And *G*_*i*_ represents the nodes set of node *i* neighborhoods. Further define the clustering coefficient of the whole network as:

(5) $$\begin{array}{l} C=\frac{1}{N}\underset{i\in G_{i}}{\sum }C\left(i\right) \end{array}$$

#### Average Weighted Degree

Average Weighted Degree is an important parameter for distinguishing networks with different topologies. The average weighted degree of the network can be obtained through averaging weights of the incident links on all the nodes in the network (Supriya et al., 2016):

(6) $$\begin{array}{l} wd=\frac{1}{N}\underset{i\in G_{i}}{\sum }s_{i} \end{array}$$

where *s*_*i*_ is described above in function (4).

#### Graph Index Complexity

Kim et al. have introduced graph index complexity as a new feature into the diagnosis of patients with AD by quantifying the complexity of the image graph (Kim and Wilhelm, 2008; Wang et al., 2016). With the largest eigenvalue of the adjacency matrix of a graph with *n* nodes presented as λ_max_ (Blinowska and Kaminski, 2013). The graph index complexity is defined as follows:

(7) $$\begin{array}{l} c_{\lambda_{\max}}=4c\left(1-c\right) \end{array}$$

where

(8) $$\begin{array}{l} c=\frac{\lambda_{\max}-2\cos\left(\pi /\left(n+1\right)\right)}{n-1-2\cos\left(\pi /\left(n+1\right)\right)} \end{array}$$

#### Degree Distribution Index

The degree distribution *P*_deg_(*k*) is often used to classify complex networks, which can be formed by counting how many nodes have each degree. In this paper, a probability distribution object is obtained by fitting the Poisson distribution to the degree distribution vector. The degree distribution *P*_deg_(*k*) is defined as

(9) $$\begin{array}{l} P_{\deg}\left(k\right)=\frac{\lambda^{k}}{k!}e^{-\lambda } \end{array}$$

The degree distribution index is defined as the λ values of the fitting distribution (Stephen and Toubia, 2009).

#### Network Entropy

The network entropy can be computed straightforwardly based on the degree distribution as

(10) $$\begin{array}{l} S=-\sum_{k}P_{\deg}\left(k\right)\log P_{\deg}\left(k\right) \end{array}$$

#### Modularity

Modularity is a quality feature that can measure the quality of the clusters (communities), which are obtained by dividing the network partition (Supriya et al., 2016). The modularity *Q* of this weighted network is defined as:

(11) $$\begin{array}{l} Q=\frac{1}{2m}\underset{i,j}{\sum }\left(a_{i,j}-\frac{k_{i}k_{j}}{2m}\right)\delta \left(C_{i},C_{i}\right) \end{array}$$

where $m=\frac{1}{2}\sum_{i,j\in G}w_{i,j}$ is the sum weights of all links in the network, $k_{i}=\sum_{j\in G}w_{i,j}$ is the sum weight of the links attached to node *i*, *C*_*i*_ represents the community which vertex *i* is assigned to, the function δ(*C*_*i*_, *C*_*j*_) is 1 if nodes *i* and *j* belong to the same community and 0 otherwise. In this paper, we used the Louvain method (Blondel et al., 2008) to distribute nodes into different communities. This method is divided into two steps. In the first step, each node is added into the neighbor communities to determine the one which can maximize the modularity gain Δ*Q*. In second step, a new network is reconstructed whose node is defined as the small community found in the first step, and whose weights of new links are given by the sum weight of the links between nodes in the corresponding two old communities. Those two steps will be repeated iteratively until the maximum of modularity is accomplished and there is no more movement of nodes. The modularity gain Δ*Q* is defined as (Zhaohong et al., 2013):

(12) $$\begin{array}{l} \Delta Q=\left[\frac{\Sigma_{\text{in}}+k_{i,\text{in}}}{2m}-{\left(\frac{\Sigma_{\text{tot}}+k_{i}}{2m}\right)}^{2}\right]\\ \;-\left[\frac{\Sigma_{\text{in}}}{2m}-{\left(\frac{\Sigma_{\text{tot}}}{2m}\right)}^{2}-{\left(\frac{k_{i}}{2m}\right)}^{2}\right] \end{array}$$

where Σ_in_ represents the sum of all the links weights inside community *C*, Σ_tot_ is the sum of the weights of the links attached to nodes in *C*, *k*_*i*_ is the sum of the weights of the links attached to node *i*, *k*_*i*,in_ is the sum of the weights of the links from *i* to nodes in *C*, and *m* is the sum weights of all links in the network.

#### Local Efficiency

Local efficiency, as a node-specific measure, is defined to measure the density of the subnetwork composed of the neighborhood of the node *i*. Local efficiency of *i*th node is given as

(13) $$\begin{array}{l} E_{loc}\left(i\right)=\frac{1}{N_{G_{i}}\left(N_{G_{i}}-1\right)}\underset{i,j\in G,i\neq j}{\sum }l_{i,j} \end{array}$$

Where *l*_*i,j*_ is the shortest distance between *i* and *j*, and *N*_*G*_*i*__ is the number of the neighborhood of node *i*. Local network efficiency is the average of the local efficiency of all nodes

(14) $$\begin{array}{l} E_{loc}=\frac{1}{N}\underset{i}{\sum }E_{loc}\left(i\right) \end{array}$$

#### Average Path Length

Average path length is a vital index to measure information transmission ability of networks. It can be used to evaluate the connectivity of the global functional network, including local and remote connection. The average path *L* is defined as:

(15) $$\begin{array}{l} L=\frac{1}{N\left(N-1\right)}\underset{i,j,i\neq j}{\sum }l_{i,j} \end{array}$$

### TSK Fuzzy Model

Given an original input dataset **X** = {**x**_1_, **x**_2_, …, **x**_*n*_} ∈ **R**^*d*^ and the corresponding class label **Y** = {**y**_1_, **y**_2_, ..., **y**_*n*_} (*y*_*i,j*_ = 1 when the *i*th sample belongs to *j*th class; otherwise, *y*_*i,j*_ = 0), the *k*th fuzzy inference rules are often defined as

$$\begin{array}{l} R^{k}:\text{IF }x_{1}\text{ is }A_{1}^{k}\wedge x_{2}\text{ is }A_{2}^{k}\wedge \ldots \wedge x_{d}\text{ is }A_{d}^{k},\text{THEN}\\ \;f_{k}\left(\text{x}\right)=\beta_{0}^{k}+\beta_{1}^{k}x_{1}+...+\beta_{d}^{k}x_{d},\text{k}=1,...,\text{K} \end{array}$$

Where $\text{x}={\left[x_{1},x_{2},...,x_{d}\right]}^{T}$ is input vector of each rule, *K* is the number of fuzzy rules, $A_{i}^{k}$ are Gaussian antecedent fuzzy sets subscribed by the input variable *x*_*i*_ of Rule *k*, ∧ is a fuzzy conjunction operator, *f*_*k*_(**x**) is a linear function about the inputs, and $\beta_{i}^{k}$ are linear parameters.

With each rule is premised on the sample vector **x**, the output of a TSK fuzzy system is expressed as

(16) $$\begin{array}{l} ỹ=\overset{K}{\underset{k=1}{\sum }}\frac{\mu_{k}\left(\text{x}\right)f_{k}\left(\text{x}\right)}{\sum_{k^{\prime }=1}^{K}\mu_{k^{\prime }}\left(\text{x}\right)}=\overset{K}{\underset{k=1}{\sum }}{\tilde{\mu }}_{k}\left(\text{x}\right)f_{k}\left(\text{x}\right) \end{array}$$

where

(17) $$\begin{array}{l} \mu_{k}\left(\text{x}\right)=\underset{i=1}{\overset{d}{\prod }}\mu_{A_{i}^{k}}\left(x_{i}\right) \end{array}$$

is the fuzzy membership function and

(18) $$\begin{array}{l} {\tilde{\mu }}_{k}\left(\text{x}\right)=\frac{\mu_{k}\left(\text{x}\right)}{\sum_{k^{\prime }=1}^{K}\mu_{k^{\prime }}\left(\text{x}\right)} \end{array}$$

is the normalized fuzzy membership function of the antecedent parameters of the kth fuzzy rule. While $\mu_{A_{i}^{k}}\left(x_{i}\right)$ is Gaussian membership function for fuzzy set $A_{i}^{k}$ that can be expressed as

(19) $$\begin{array}{l} \mu_{A_{i}^{k}}\left(x_{i}\right)=\exp\left(-\frac{{\left(x_{i}-c_{i}^{k}\right)}^{2}}{\delta_{i}^{k}}\right) \end{array}$$

where $c_{i}^{k}$ is kth cluster center parameters, which can be calculated with the classical fuzzy c-means (FCM) clustering algorithm (Bezdek et al., 1984):

(20) $$\begin{array}{l} c_{i}^{k}=\frac{\overset{N}{\underset{j=1}{\sum }}u_{jk}x_{ji}}{\overset{N}{\underset{j=1}{\sum }}u_{jk}} \end{array}$$

and the width parameter $\delta_{i}^{k}$ can be estimated by (Zhaohong et al., 2013):

(21) $$\begin{array}{l} \delta_{i}^{k}=\frac{h\cdot \overset{N}{\underset{j=1}{\sum }}u_{jk}{\left(x_{ji}-c_{i}^{k}\right)}^{2}}{\overset{N}{\underset{j=1}{\sum }}u_{jk}} \end{array}$$

where the element *u*_*jk*_ ∈ [0, 1] denotes the fuzzy membership of *n*th input sample **x**_*n*_ to the *k*th cluster (*k* = 1, 2, ..., *K*), *h* is a constant called the scale parameter.

For an input sample **x**_*n*_, let

(22) $$\begin{array}{l} {\text{x}}_{n,e}={\left(1,{{\text{x}}_{n}}^{T}\right)}^{T} \end{array}$$

(23) $$\begin{array}{l} {\tilde{x}}_{n}^{k}={\tilde{\mu }}^{k}\left({\text{x}}_{n}\right){\text{x}}_{e} \end{array}$$

(24) $$\begin{array}{l} \rho \left({\text{x}}_{n}\right)={\left({\left({{\tilde{x}}_{n}}^{1}\right)}^{T},{\left({{\tilde{x}}_{n}}^{2}\right)}^{T},...,{\left({{\tilde{x}}_{n}}^{K}\right)}^{T}\right)}^{T}\in R^{K\left(d+1\right)} \end{array}$$

(25) $$\begin{array}{l} \beta^{k}={\left(\beta_{0}^{k},\beta_{1}^{k},...,\beta_{d}^{k}\right)}^{T} \end{array}$$

(26) $$\begin{array}{l} \beta_{g}={\left({\left(\beta^{1}\right)}^{T},{\left(\beta^{2}\right)}^{T},...,{\left(\beta^{K}\right)}^{T}\right)}^{T} \end{array}$$

then the output value ỹ_*n*_ of a TSK fuzzy classifier for sample **x**_*n*_ can be expressed as

(27) $$\begin{array}{l} {ỹ}_{n}={\beta_{g}}^{T}\rho \left({\text{x}}_{n}\right) \end{array}$$

### Learning Algorithm

Given a training dataset $D_{S}=\left\{{\text{x}}_{i},{\text{y}}_{i}|{\text{x}}_{i}\in R^{d},{\text{y}}_{i}\in R^{C},i=1,...,N_{S}\right\}$, where *C* is the number of classes, the consequent parameter β_*g*_ can be learned by using generalized hidden-mapping ridge regression (GHRR) (Deng et al., 2014; Tian et al., 2019). The objective function is:

(28) $$\begin{array}{l} \underset{\beta_{g}}{\min}J\left(\beta_{g}\right)=\frac{1}{2}\overset{C}{\underset{j=1}{\sum }}{\overset{N_{S}}{\underset{i=1}{\sum }}\|{\beta_{g,j}}^{T}{\text{x}}_{g,i}-y_{i,j}\|}^{2}+\frac{\lambda }{2}\overset{C}{\underset{j=1}{\sum }}{\beta_{g,j}}^{T}\beta_{g,j} \end{array}$$

where is the consequent parameter vector of the *j*th class is represented by β_*g,j*_, λ is a regularization parameter controls the complexity of the classifier, and the tolerance of error λ can be set manually or determined by cross-validation. The optimal consequent parameters, β_*g,j*_, can be computed by setting the derivatives of *J* with regard to each β_*g,j*_ is 0 and the solution is (Yu et al., 2020):

(29) $$\begin{array}{l} \beta_{g,j}={\left(\lambda_{1}{\text{I}}_{\left(d+1\right)*K\times \left(d+1\right)*K}+\overset{N_{S}}{\underset{i=1}{\sum }}{\text{x}}_{g,i}{{\text{x}}_{g,i}}^{T}\right)}^{-1}\overset{N_{S}}{\underset{i=1}{\sum }}{\text{x}}_{g,i}y_{i,j} \end{array}$$

## Experimental Results

**Figure 2:**
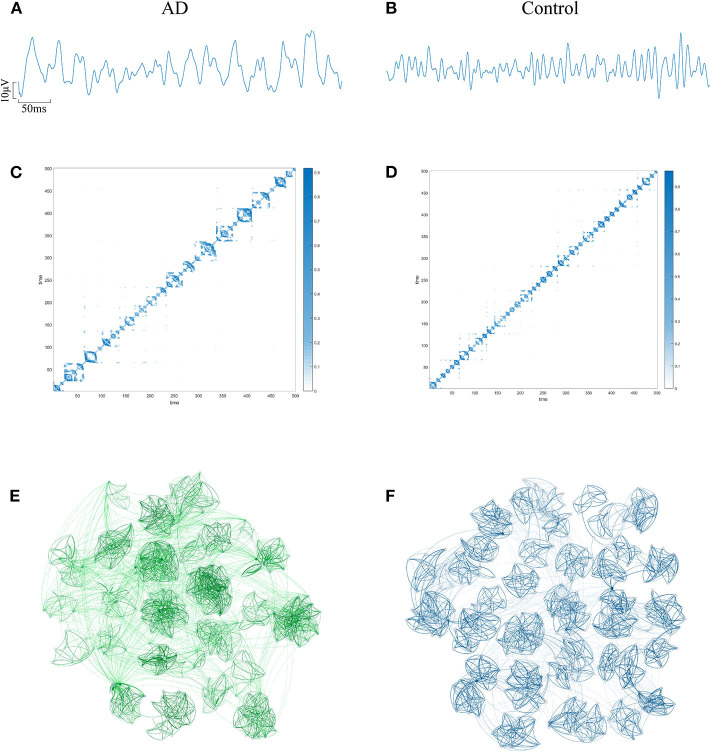
An example of converting EEG signal from an AD subject and a control subject into a WVG. EEG signals of FP1 channel from AD **(A)** subject and control **(B)** subject with 5s length. The adjacency matrixes of the converted WVGs respectively for AD **(C)** subject and control **(D)** subject. Schematic diagram of complex networks corresponding to WVGs of AD **(E)** subject and control **(F)** subject.

The EEG of AD patients implies a large amount of information that cannot be visually expressed from the waveform. Research shows that the visualization algorithm can express the hidden information in the form of images. In order to verify whether the AD brain's electrical features can be represented by WVG, we first select the same channel EEG from an AD patient and a control subject. Two episodes with a length of 500 data points (as shown in Figures 2A,B) are further intercepted, and converted to WVG. The result is shown in Figures 2C,D. Studies have reported that it's easy to detect a diffuse slowing in the EEG of AD patients with the naked eye (Micanovic and Pal, 2014). This diffuse slowing feature is well-preserved in WVG, and WVG of AD patients can be clearly observed in more communities, indicating the feasibility of WVG method for AD detection. For further observation of the topological feature of the WVG network, the two adjacency matrixes are represented as network structure diagrams that shown in Figures 2E,F. The dots in figure represent all network nodes and the network edges are represented by curves, and the shade of the curve color can directly reflect the weighted value of the edges. It can be observed that the different communities in the WVG network of normal people are generally similar in size and the distributions of connections are uniform. The community structure of the networks obtained by the WVG method is more irregular for AD patients. Most nodes are concentrated in a small part of communities, and the connection between communities is also closer. The result indicates that the electrophysiological signals of AD brains are more unstable, with fluctuations that are stronger. Research on single channel reveals that the WVG network of AD and normal people are significantly difference. Next, we will transform all 16 channels into multi-networks ({*y*_*n*_}(1 ≤ *n* ≤ 16)) and each layer of network can be obtained from each channel. We further considered which parameters are selected to quantify this difference.

**Figure 3:**
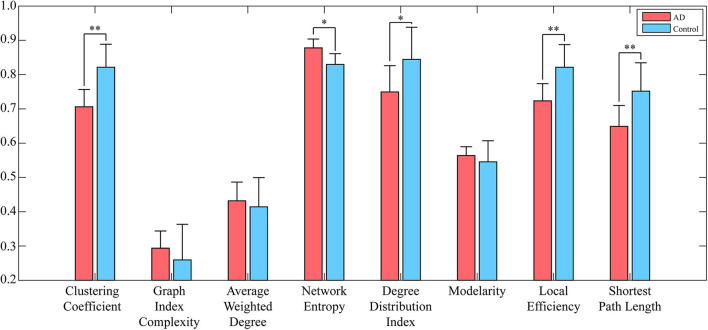
Network parameters (averaged across subjects) of both AD networks and control networks. Error bars represent standard error across subjects. The degree of significant difference is calculated by Analysis of Variance (ANOVA) across all subjects. ^**^A significant correlation (*p*_*c*_ ≤ 0.01 corrected for multiple comparisons across tiles). ^*^A trend (*p*_*c*_ ≤ 0.05).

To reduce the computing time and to retain as much information as possible, the EEG signal is divided into many episodes through sliding windows with lengths of 500 data points. Since the size of the converted WVG network is consistent with the length of EEG series, a series of adjacency matrixes of size 500 × 500 are finally obtained. Next, we calculate clustering coefficient (*x*_1_), graph index complexity (*x*_2_), average weighted degree (*x*_3_), network entropy (*x*_4_), degree distribution index (*x*_5_), modularity (*x*_6_), local efficiency (*x*_7_), and average path length (*x*_8_) of each WVG network of both AD and control. Above parameters can be obtained from each different network layers, which can be considered as different features. Since there is a considerable difference in the magnitude of the values of different parameters, the calculated result is normalized to 0~1. All windows of each person were further averaged, and then a statistical analysis was performed based on each person. As shown in Figure 3, parameters of all subjects are statistically analyzed and the parameters that are significantly different for AD group and control group are marked with ^*^. The values of clustering coefficient, local efficiency, and shortest path length of the AD group are significantly lower than that of controls with *p* < 0.01. Meanwhile, the degree distribution entropy of AD group is higher than that of controls with *p* < 0.05 while the degree distribution lambda of AD group is lower than that of controls with *p* < 0.05.The obtained results demonstrate that network topological parameters can be used to detect AD.

Through statistical analysis, it's obvious that some of the above parameters can clearly distinguish AD from the control group. In order to further verify the effect of these parameters on AD recognition, these parameters will be used as input features of the training fuzzy classifier. In each training process, we randomly select 80% of the original data to form training datasets which can be used for ten-fold cross-validation (10-CV), with 90% (90% × 80%) utilized for model training and 10% (10% × 80%) for constructing a validation set. The above procedure is repeated 10 times to cover the entire training set and finally determine the optimal hyperparameters of the TSK model. The remaining 20% of all data is tested as the testing data with determined hyperparameters. For each different input feature or feature vector, the classification results (accuracy, sensitivity, specificity) are averaged after training for 50 times.

The construction of each WVG network is based on a single time series, so 16 WVGs are obtained from 16-channel EEG used in this paper. These WVG networks contain different electrophysiological information of neurons in different brain regions. However, in the existing studies, the parameters extracted from WVG networks constructed by different brain regions' EEG were usually regarded as the same class of features, so the differences between brain regions were ignored. Therefore, we consider the 16 WVG networks as different networks and combine them into a multi-layer network. In order to verify whether the underlying dynamic information of these network layers are different, the classification is first performed with a single feature as input. Each parameter extracted from each single network layer transformed from different channels is used as the single input feature for model training, and the classification results are shown in Table 1 with optimal classification result is bolded. It can be observed that for the same network parameter extracted from different network layers, the classification results are significantly different. The difference in classification accuracy of the same parameter from different network can even reach 28.39% for average weighted degree ({(*x*_3_, *y*_*k*_)}, *k* = 1, ..., 16), indicating that the dynamic information that contained in EEG of different brain regions does have significant differences and parameters of different layers maybe independent from each other. This finding shows that the network characteristics of the multi-network composed of WVG network layers can be used as independent input features for the classifier.

**Table 1:** Classification results with each single parameter from single network layer is taken as input feature.

	**x_**1**_**	**x_**2**_**	**x_**3**_**	**x_**4**_**	**x_**5**_**	**x_**6**_**	**x_**7**_**	**x_**8**_**
y_1_	0.6218	0.5546	0.5618	0.6543	0.5654	0.5457	0.5721	0.5904
y_2_	0.5925	0.6550	0.5086	0.6368	0.5275	0.5364	0.5721	0.6893
y_3_	0.6225	0.5482	0.5454	0.6454	0.6214	0.5596	0.5996	0.5243
y_4_	0.6718	0.4779	0.5514	0.5632	0.6464	0.5229	0.5800	0.6146
y_5_	0.5754	0.5918	0.5507	0.6343	0.5443	0.5382	0.5868	0.5982
y_6_	0.6600	0.5718	0.5582	0.5486	0.5536	0.5625	0.5675	0.6386
y_7_	0.5721	0.5793	0.5086	0.7893	0.6211	0.5779	0.5964	0.5293
y_8_	0.5461	0.5550	0.6279	0.5957	0.6207	0.5679	0.5818	0.5679
y_9_	0.7925	0.6671	0.6446	0.54	0.5454	0.7446	0.7182	0.5768
y_10_	0.5271	0.3804	0.5811	0.6625	0.5504	0.5693	0.5225	0.5479
y_11_	0.5157	0.425	0.5304	0.5107	0.5257	0.5275	0.5179	0.5075
y_12_	0.6996	0.6832	0.6193	0.7432	0.6789	0.7904	0.745	0.7386
y_13_	**0.7996**	0.6411	0.5664	0.5586	0.6325	0.7218	0.69	0.5343
y_14_	0.5279	0.515	0.5082	0.5218	0.5314	0.5464	0.5379	0.5107
y_15_	0.5625	0.4011	0.5071	0.5529	0.365	0.6868	0.3814	0.3789
y_16_	0.7489	0.5479	0.5082	0.5486	0.54	0.6979	0.6175	0.4589

The input feature vector consisting of multiple parameters is used for fuzzy system training. The classification will be performed based on the following three feature sets [as shown in Figure 1(3)]: (1) Single parameter from multi-networks: When ensuring that the classifier input is the same parameter, select different network layers for parameter extraction and combination. (2) Multi-parameters from single network: In the case of one single network layer, different parameters are extracted and selected for combination as a classifier input. (3) Multi-parameters from multi-network: All parameters extracted from all network layers are used as different input features to the classifier. Then for each set, various feature select methods including Correlation-based Feature Selection (CFS) (Guyon et al., 2002), Dependence Guided Unsupervised Feature Selection (DGUFS) (Zhu et al., 2017), Fisher (Gu et al., 2012), Feature Selective Validation (FSV) (Bradley and Mangasarian, 1999), Locality-Constrained Linear Coding Feature Select (LLCFS) (Zeng and Cheung, 2011), and minimum-redundancy maximum-relevance (mRMR) (Peng et al., 2005) are used to sort the features to obtain the feature sequence for each set. According to the obtained feature sequence, select the different number of features in order (i.e., the first one feature, the first two features, the first three features.) to component the input vectors for the TSK model training process. In the feature select process (as shown in Figure 1), the methods of Feature Selection Library (FSLib) are adopted for determining feature input vectors of TSK. All the algorithms are implemented with MATLAB 2016b.

First, case 1 is described as an example, and the structure of TSK is also described in details in following. As the clustering coefficient ({(*x*_1_, *y*_13_)}) reached a highest accuracy of 79.96% in Table 1, local efficiency from all network layers ({(*x*_1_, *y*_*k*_)}, *k* = 1, ..., 16) is adopted for feature selection and multi-input classification. The orders of the features are obtained by various sorting feature selection algorithms. After the ranking of network parameters, we choose input feature vectors with different lengths as inputs of TSK model and calculate classification results (accuracy, sensitivity, and specificity), respectively. The optimal length of input vectors and classification results are shown in Table 2. It can be observed that with different feature select methods, the length of the feature vectors with the optimal classification result is different. Besides, the sensitivity is higher than the specificity for the feature vectors filtrated by CFS and DGUFS methods, while the others are opposite. It shows that the change of the feature used for training will affect the properties of the trained model. As for the parameter set of clustering coefficients extracted from multiple networks, the Fisher method can be used to achieve the optimal classification result. The classification process with Fisher method are further explored.

**Table 2:** Classification results with the set of single parameter from multiple networks is taken as input feature vector.

**Method**	**Best input**	**Accuracy**	**Sensitivity**	**Specificity**
	**vector length**			
CFS	4	0.9211	0.9375	0.9091
DGUFS	3	0.7971	0.8974	0.6667
Fisher	4	0.9528	0.9491	0.9583
FSV	6	0.9430	0.9406	0.9449
LLCFS	9	0.9431	0.9223	0.9600
mRMR	6	0.8706	0.7971	0.9091

**Figure 4:**
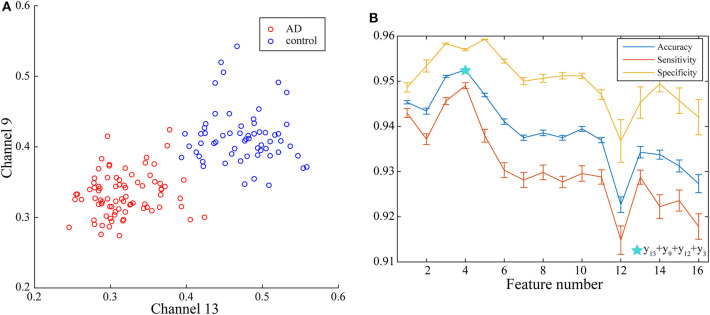
**(A)** Joint distribution of clustering coefficient obtained from WVG network transformed from Channel 13 and Channel 9. **(B)** Classification results when the number of input features is from 1 to 16, which is obtained under single parameter (clustering coefficient) from multi-networks set and ordered through feature selection method.

With the applying of Fisher algorithm, the order of the parameters is obtained as (*x*_1_, *y*__13__), (*x*_1_, *y*_9_), (*x*_1_, *y*__12__), (*x*_1_, *y*__3__), (*x*_1_, *y*_1_), (*x*_1_, *y*_2_), (*x*_1_, *y*_1_), (*x*_1_, *y*_6_), (*x*_1_, *y*__10__), (*x*_1_, *y*_8_), (*x*_1_, *y*_5_), (*x*_1_, *y*__15__), (*x*_1_, *y*__7__), (*x*_1_, *y*__4__), (*x*_1_, *y*_14_), (*x*_1_, *y*__6__). The joint distribution of the first two channels under the ranking is illustrated to verify the effectiveness of the same network parameter of WVG network transformed from different channels as the multi-input for classification. The result is shown in Figure 4A with each point represents a subject. It's obviously that AD subjects display significant differences from controls, which also demonstrate that local efficiencies, respectively, of channel 9 and channel 13 are effective to classify AD and controls. These two parameters can also get the best classification results when multi-network clustering coefficient is taken as single parameter input. However, the optimal parameters obtained by feature selection are not completely consistent with those that are optimal for the classification result when a single parameter is used as input. This indicates that the information of a single brain region cannot be used as a direct feature to distinguish patients with AD, but the implicit information of different brain regions can complement each other. In the above ranking order, five rules TSK classifiers are used with the number of classifier inputs is from 1 to 16 in order, and the final classification results under cross-validation are listed in Figure 4B. As the length of input feature vector increases, the accuracy reaches a maximum of 95.28% at four inputs and then begins to decrease.

In this part, the framework of the TSK is also described in details based on the selected optimal combination feature. The input vector **x** consists of the clustering coefficients of channel 13((*x*_1_, *y*__13__)), channel 9((*x*_1_, *y*_9_)), channel 12((*x*_1_, *y*__12__)), and channel 3((*x*_1_, *y*__3__)). Membership functions can be linguistically expressed using a fuzzy linguistic description including “*very low*,” “*low*,” “*medium*,” “*high*,” and “*very high*.” Each membership function of different features corresponds to different description in ascending order of the values of centers. To provide further explanation, the clustering coefficient of channel 13 is interpreted as an example. We define the gaussian model as a membership function, and each rule will get a set of antecedent parameter (centers, standard variance), respectively, which are (0.3990 0.0031) for Rule 1, (0.3956 0.0030) for Rule 2, (0.4165 0.0032) for Rule 3, (0.4052 0.0031) for Rule 4, and (0.4040 0.0030) for Rule 5. By the permutation of these five centers of each rule, membership functions can be described with fuzzy linguistic description: Rule 1 is “*very low*,” Rule 2 is “*very high*,” Rule 3 is “*low*,” Rule 4 is “*medium*,” and Rule 5 is “*high*.” The other four features can also be fuzzy and described similarly. Therefore, with the linguistic expressions and the corresponding linear function the fuzzy rule can be given as follows:

*R*^1^ : IF*y*_13_ is ***very low*** ∧ *y*_9_ is ***very low*** ∧ *y*_12_ is ***medium*** ∧ *y*_3_ is ***very low***,

$$\begin{array}{l} \text{THEN }f_{1}\left(x\right)=\\ \;\left[\begin{array}{c} 0.4975-0.1872y_{13}+0.1615y_{9}+0.1515y_{12}+0.2134y_{3}\\ -0.1385-0.0959y_{13}-0.0738y_{9}-0.0514y_{12}-0.1147y_{3} \end{array}\right], \end{array}$$

*R*^2^ : IF*y*_13_ is ***very high*** ∧ *y*_9_ is ***very high*** ∧ *y*_12_ is ***very low*** ∧ *y*_3_ is ***very high***,

$$\begin{array}{l} \text{THEN }f_{2}\left(x\right)=\\ \;\left[\begin{array}{c} -1.99\text{e-}4+1.59\text{e-}5y_{13}-9.79\text{e-}5y_{9}-2.13\text{e-}5y_{12}+2.03\text{e-}4y_{3}\\ 0.0013+3.34\text{e-}4y_{13}+4.12\text{e-}4y_{9}+2.65\text{e-}4y_{12}+1.15\text{e-}4y_{3} \end{array}\right], \end{array}$$

*R*^3^ : IF*y*_13_ is ***low*** ∧ *y*_9_ is ***low*** ∧ *y*_12_ is ***high*** ∧ *y*_3_ is ***low***,

$$\begin{array}{l} \text{THEN }f_{3}\left(x\right)=\\ \;\left[\begin{array}{c} 0.2508-0.0485y_{13}+0.0555y_{9}+0.0167y_{12}+0.1049y_{3}\\ -0.0039-0.0195y_{13}-0.0095y_{9}-0.0572y_{12}-0.0341y_{3} \end{array}\right], \end{array}$$

*R*^4^ : IF*y*_13_ is ***medium*** ∧ *y*_9_ is ***high*** ∧ *y*_12_ is ***very high*** ∧ *y*_3_ is ***medium***,

$$\begin{array}{l} \text{THEN }f_{4}\left(x\right)=\\ \;\left[\begin{array}{c} -\text{0.0536}-\text{0.0429}y_{13}-\text{0.0341}y_{9}-\text{0.0765}y_{12}-2.05\text{e-}4y_{3}\\ 0.2071+0.0872y_{13}+0.0759y_{9}+0.1244y_{12}+0.0450y_{3} \end{array}\right], \end{array}$$

*R*^5^ : IF*y*_13_ is ***high*** ∧ *y*_9_ is ***medium*** ∧ *y*_12_ is ***low*** ∧ *y*_3_ is ***high***,

$$\begin{array}{l} \text{THEN }f_{5}\left(x\right)=\\ \;\left[\begin{array}{c} 0.0130+0.0016y_{13}-0.0020y_{9}+0.0025y_{12}+0.0041y_{3}\\ 2.47\text{e-}4+0.0026y_{13}+0.0017y_{9}+0.0011y_{12}+4.33\text{e-}5y_{3} \end{array}\right]. \end{array}$$

The fuzzy system that has been learned based on these five rules above, the example with an input of [0.2098 0.2106 0.3585 0.2264] is given to further explain the mechanism of testing process. Inputs of the identification process based on the trained fuzzy system are the network features of an AD patient, and the decision output is the prediction of label vector. The sum of the five calculated rule-based outputs is *f* = [0.8940 0.00956]^*T*^, then the maximal element in *f* is set to 1 while others to 0 for handling the decision output. Finally, AD patient can be identified based on the final value of the output *y* = [1 0]^*T*^.

**Figure 5:**
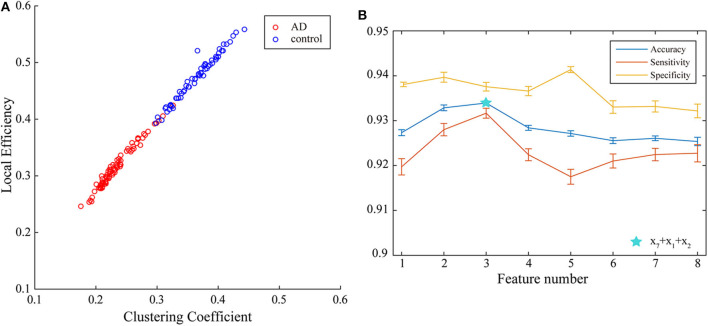
**(A)** Joint distribution of clustering coefficient and local efficiency obtained from WVG network obtained from Channel 13. **(B)** Classification results when the number of input features is from 1 to 8, which is obtained under multi-parameters from single network (*y*_13_) set and ordered through feature selection method.

Next, multi-parameters from single network are also used as input set for the classifier together. The classification results obtained by various feature select methods and the optimal lengths of input feature vectors are shown in Table 3. The parameters selected by FSV method can be used to form the vector to obtain the optimal classification result, and the sorted parameters are further analyzed in detail. The features in order obtained through the FSV algorithm is (*x*_7_, *y*_13_), (*x*_1_, *y*_13_), (*x*_2_, *y*_13_), (*x*_3_, *y*_13_), (*x*_8_, *y*_13_), (*x*_5_, *y*_13_), (*x*_6_, *y*_13_), (*x*_4_, *y*_13_). Clustering coefficients ((*x*_7_, *y*_13_)) and local efficiencies ((*x*_1_, *y*_13_)) are chosen to verify the feasibility of the classification, and the image is shown in Figure 5A. It is clear that there is a significant difference between the AD and the control group. The TSK classification is applied to all feature input groups. As shown in Figure 5B, the classification accuracy reaches a maximum value of 93.41% when the first three features are taken as input vector. The optimal combination obtained by the feature sorting method is local efficiency (*x*_7_), clustering coefficient (*x*_1_), and graph complexity index (*x*_2_). The graph complexity index has a low discrimination between AD and the control group, and the TSK models trained with graph complexity index extracted from each network layer as single input have low classification accuracy. However, the image complexity index can supplement the clustering coefficient and local efficiency, indicating that the redundancy between some parameters from same network layer is small, which is of great significance as a feature of model training. Through the above classification results, multi-parameters, and multi-networks can both be applied to the TSK classification, and they are not the same type as input sets for model training.

**Table 3:** Classification results with the set of multiple parameters from single network is taken as input feature vector.

**Method**	**Best input**	**Accuracy**	**Sensitivity**	**Specificity**
	**vector length**			
CFS	3	0.9259	0.9434	0.9091
DGUFS	4	0.8734	0.8772	0.8696
Fisher	2	0.8969	0.9259	0.8696
FSV	3	0.9341	0.9379	0.9321
LLCFS	6	0.9009	0.9091	0.8929
mRMR	5	0.7937	0.7692	0.8197

**Figure 6:**
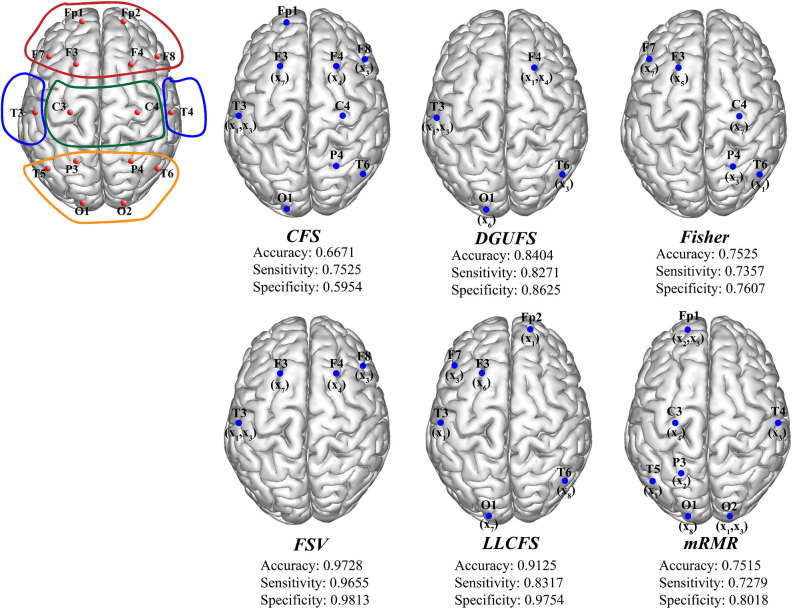
The schematic diagram of channel position with the frontal lobe is marked within red lines, the temporal lobe in blue, the parietal lobe in green, and the occipital lobe in orange. The optimal parameters and corresponding classification results under different feature select methods.

Finally, the multi-parameters from multi-networks are used for training. We further applied different feature select methods on this input set, and find the best feature input vectors, respectively. Figure 6 provides the methods and corresponding classification results. The brain area enclosed by the red line is the frontal lobe, the blue is the temporal lobe, the green is the parietal lobe, and the orange is the occipital lobe. It can be observed that the parameters that are filtered by different methods are more common to be extracted from the network layers of the frontal EEG. This suggests that information in the frontal lobe is more effective in identifying AD patients. Damage to the frontal lobe of the brain, which plays a prominent role in thinking and behavior, can lead to forgetfulness, delayed behavior, and distraction. Meanwhile, signals from other brain regions also play an important role in AD recognition, indicating that AD disease has a global impact on the brain. The best result of multi-parameters from multi-networks set are selected through FSV method, which up to 97.28%. The combination is {(*x*_7_, *y*_3_), (*x*_3_, *y*_13_), (*x*_1_, *y*_13_), (*x*_3_, *y*_12_), (*x*_4_, *y*_4_)}. The accuracy rate with set 3 is improved compared with set 1 and set 2, indicating that it is of certain significance to take multiple parameters extracted from multiple networks as different features.

## Conclusion and Discussion

This paper proposes a multi-input machine learning method that combines fuzzy classifier and WVG to identify AD patient's EEG. In order to improve the interpretability and recognition accuracy of the model, complex network theory and TSK fuzzy system model is adopted. A WVG network layer is constructed using a single channel EEG. The multi-parameters obtained from multiple networks can be used as independent input features for model training, and the TSK model based on fuzzy rules is used to classify AD EEG with better interpretability. We considered three types of classification input sets: multi-parameters from single network, single parameter from multi-networks, and multi-parameters from multi-networks. These three types of inputs are, respectively, applied as the training set of the learning of the TSK model. The experimental results show that the fuzzy model-based system model can achieve optimal performance with multi-parameters from multi-networks as classification input set, and the accuracy is up to 97.83%. Meanwhile, the optimal input numbers are different for the three types of input sets proposed in this paper. The best input combination is 5 input features in the input set of multi-parameters from multi-networks.

The current clinical techniques of AD identification, mainly including the scale assessing, cerebrospinal fluid examination, and the observation of atrophy of gray matter through the brain functional imaging, are difficult to obtain reliable diagnostic markers. It is also difficult to find obvious organic changes in the early stage of AD. We propose an AD diagnostic model that combines the TSK fuzzy model with complex network obtained by WVG method and propose three different kinds of training input sets, which provides a new method for the search of AD EEG biomarkers. Compared with traditional methods, the AD identification approach proposed in this paper, has lower implementation difficulty and higher accuracy.

EEG, which is commonly considered to have significant chaotic characteristics, cannot be well-evaluated with linear analysis. The WVG method used in this paper can transform the one-dimensional time series into images and extract the underlying information contained in electrophysiological activities of different brain regions. In contrast with other network construction methods like synchronous network, the WVG networks obtained by each EEG channel are independent of each other. Thus, more network features can be found and effective biomarkers can be obtained from kinds of feature sets with WVG (Zhu et al., 2014). The classification results show that this WVG method is very effective for feature extraction of AD recognition. In future works it will be combined with multi-layer network theory, further discussing the correlation between different channels with constructing multi-layer network. In past research we confirmed the feasibility of the multi-layer network scheme, and extracted the multiplex clustering coefficient and multiplex participation coefficient (Cai et al., 2020). Future work will consider both the implicit characteristics of single channels and the information integration between multiple channels.

We propose three different kinds of feature sets and prove that the optimal parameter vectors can be obtained from the set multi-parameters from multi-networks. This finding indicates that simultaneously considering different networks and different parameters as disparate features has obvious help for the acquisition of AD biomarkers. At the same time, the classification results show that the excessive features as input is not conducive to the optimization of the classification model, so it is necessary to reduce the feature dimension. Too much feature increase may lead to the overfitting of the learning model, and even the increase of invalid features may lead to the decrease of the accuracy based on test set (Guyon et al., 2002). Therefore, the application of feature selection plays an important role in improving the accuracy of fuzzy learning models.

In this paper, we combined the identification model combining feature selection approaches with machine learning. Researchers can effectively reduce the number of EEG channels, and the difficulty of data collection will be significantly reduced. Meanwhile, with the reduction of the parameters, it can be easier to improve the efficiency of the AD recognition process. Compared with traditional manual diagnosis, machine learning methods have higher reliability, and improved recognition accuracy. Especially, the TSK method has higher interpretability and robustness by integrating the advantages of fuzzy rules and membership functions. There are still some limitations in our research. We used a variety of feature selection methods, but a feature selection method suitable for the highly interpretable TSK model is necessary to be considered. Future work may focus on how to select features more efficiently and accurately to achieve higher classification accuracy.

## Data Availability Statement

The datasets generated for this study are available on request to the corresponding author.

## Ethics Statement

The studies involving human participants were reviewed and approved by the Ethics committee of Tangshan Gongren hospital. The patients/participants provided their written informed consent to participate in this study.

## Author Contributions

HY: article writing, design of methods, and article correction. LZ: article writing, processing and analysis of data, and design of methods. LC: design of methods and data analysis. JW: data analysis and article review. JL: data collection. RW: article review and correction. ZZ: data collection. All authors contributed to the article and approved the submitted version.

## Conflict of Interest

The authors declare that the research was conducted in the absence of any commercial or financial relationships that could be construed as a potential conflict of interest.
